# Metataxonomic Analysis of the Uterine Microbiota Associated with Low Fertility in Dairy Cows Using Endometrial Tissues Prior to First Artificial Insemination

**DOI:** 10.1128/spectrum.04764-22

**Published:** 2023-04-26

**Authors:** Takuya Yagisawa, Jumpei Uchiyama, Iyo Takemura-Uchiyama, Shun Ando, Osamu Ichii, Hironobu Murakami, Osamu Matsushita, Seiji Katagiri

**Affiliations:** a Hokkaido Agriculture Mutual Aid Association, Sapporo, Japan; b Department of Bacteriology, Graduate School of Medicine Dentistry and Pharmaceutical Sciences, Okayama University, Okayama, Japan; c Laboratory of Anatomy, Department of Basic Veterinary Sciences, Faculty of Veterinary Medicine, Hokkaido University, Hokkaido, Japan; d Laboratory of Agrobiomedical Science, Faculty of Agriculture, Hokkaido University, Hokkaido, Japan; e Laboratory of Infectious Diseases, School of Veterinary Medicine, Azabu University, Kanagawa, Japan; f Laboratory of Theriogenology, Department of Clinical Sciences, Faculty of Veterinary Medicine, Hokkaido University, Hokkaido, Japan; University of Arkansas for Medical Sciences

**Keywords:** dairy cows, low fertility, uterine microbiota, microbial diversity, bacterial association

## Abstract

The deterioration in reproductive performance in association with low fertility leads to significant economic losses on dairy farms. The uterine microbiota has begun to attract attention as a possible cause of unexplained low fertility. We analyzed the uterine microbiota associated with fertility by 16S rRNA gene amplicon sequencing in dairy cows. First, the alpha (Chao1 and Shannon) and beta (unweighted and weighted UniFrac) diversities of 69 cows at four dairy farms that had passed the voluntary waiting period before the first artificial insemination (AI) were analyzed with respect to factors including farm, housing style, feeding management, parity, and AI frequency to conception. Significant differences were observed in the farm, housing style, and feeding management, except parity and AI frequency to conception. The other diversity metrics did not show significant differences in the tested factors. Similar results were obtained for the predicted functional profile. Next, the microbial diversity analysis of 31 cows at a single farm using weighted UniFrac distance matrices revealed a correlation with AI frequency to conception but not with parity. In correlation with AI frequency to conception, the predicted function profile appeared to be slightly modified and a single bacterial taxon, *Arcobacter*, was detected. The bacterial associations related to fertility were estimated. Considering these, the uterine microbiota in dairy cows can be varied depending on the farm management practices and may become one of the measures for low fertility.

**IMPORTANCE** We examined the uterine microbiota associated with low fertility in dairy cows derived from four commercial farms via a metataxonomic approach using endometrial tissues prior to the first artificial insemination. The present study provided two new insights into the relevance of uterine microbiota with respect to fertility. First, the uterine microbiota varied depending on housing style and feeding management. Next, a subtle change was observed in functional profile analysis: a formation of uterine microbiota was detected to be different in correlation with fertility in one farm studied. Considering these insights, an examination system on bovine uterine microbiota is hopefully established based on continuous research on this topic.

## INTRODUCTION

The deterioration in reproductive performance caused by prolonged intervals from calving to conception in dairy cows is causing significant economic losses to dairy farms (1–4). The increase in the net cost of 1 day until the time to conceive is estimated to range from €0.28 to €1.10 per cow per day (1). As much as 25% of dairy cows are culled because of reproductive problems, which accounts for a higher proportion than other major factors, such as mastitis and lameness (3, 5). Low fertility is one of the major factors underlying the deterioration in reproductive performance (3, 4). The causes of low fertility vary widely, from management factors (e.g., heat detection, nutritional control, and cowshed environment) to cow-specific factors (e.g., reproductive tract infections, endocrine disorders, and defective ova) (6, 7). However, some cases remain unexplained by these factors, resulting in losses to farms without adequate countermeasures (6). Thus, the study of factors potentially associated with low fertility is important, because it may lead to the development of new measures for reproductive management.

The uterine microbiota has recently gained interest in this field of research. Although the uterus has been thought of as being germfree for decades, the advances in next-generation sequencing technology have revealed the existence of uterine microbiota; contrarily, the microbiota that otherwise cannot be isolated using conventional bacterial culture techniques (8, 9). Pioneer studies on the relationship between the uterine microbiota and fertility have been performed in humans (10). Depending on the type of bacteria present in the uterine microbiota, positive and negative relationships with reproductive function have been shown (10). Studies comparing the compositions of the uterine microbiota of pregnant and nonpregnant women have revealed that the presence of *Lactobacillus*-dominated microbiota is associated with significantly increased pregnancy rates (11–13), whereas higher proportions of *Atopobium* spp., *Bifidobacterium* spp., *Gardnerella* spp., *Klebsiella* spp., and *Streptococcus* spp. cause inflammation in the reproductive tract and are considered to be negatively associated with fertility (14, 15). Thus, the uterine microbiota can be one of the possible factors related to conception in humans.

The concerns regarding the uterine microbiota throughout the process from postpartum uterine involutions to conception by artificial insemination (AI) are increasing in cows as well. To facilitate reproductive management, each farm sets a voluntary waiting period (VWP) between calving and first insemination to ensure sufficient time for uterine involution to prepare for the next pregnancy. In dairy cows, generally, the VWP is set at 60 days after parturition (16–18). The uterine microbiota of cows is presently being studied with respect to the relations to postpartum uterine inflammation (19–21). Increased relative abundances of *Bacteroidetes* and *Fusobacterium* and decreased relative abundances of *Proteobacteria* and *Tenericutes* are reported to be significantly associated with postpartum uterine inflammation, resulting in delayed uterine involutions (19–21). Conversely, few studies are available on the uterine microbiota associated with fertility after the VWP, despite its importance in understanding the causes of unexplained low fertility (22, 23). The relationship between conception rate and uterine microbiota within timed AI sampled by a uterine lavage approach in beef cows has been recently examined, and a lower microbial diversity with specific bacterial genera is found in nonpregnant cows compared with that in pregnant cows (23). To evaluate the relationship between the uterine microbiota and fertility clinically, cows must be monitored until conception is confirmed (2–4). Because the AI frequency to conception can be used as a measure of fertility (6), the relationship between the uterine microbiota and AI frequency to conception is worth investigating.

Endometrial tissue biopsies enable the detection of bacteria not only on the surface layer of mucus, inside the mucus layer, but also on the endometrium (24–26). In this study, the uterine microbiota associated with low fertility was investigated via a metataxonomic approach using endometrial biopsy samples from dairy cows after the VWP, before the first insemination.

## RESULTS

### Uterine microbial community analysis.

The microbiota data using 16S rRNA amplicon sequencing were collected from endometrial tissue biopsy samples. Sixty-nine cows bred on four commercial dairy farms were enrolled in this study (see Fig. S1 in the supplemental material). From the clinical point of view, cows that do not conceive within the first three inseminations are clinically recognized as having low fertility (6). The rates of low-fertility cows (≥4 AI) ranged from 27 to 39% among the farms. The information on the dairy farms is provided in Table 1.

**TABLE 1:** Information about the four farms

Farm	Herd size (head)	Housing style	Feeding management^*a*^	No. of cows studied
F	70	Tie stall barn	Separate feeding	13
ND	50	Tie stall barn	TMR_B	11
NG	144	Free barn	TMR_A	31
S	65	Tie stall barn	TMR_B	14

a TMR_A, total mixed ration (TMR) supplied at own farm; TMR_B, TMR purchased from the TMR center, which is a cooperative organization of farmers who supply TMR feed from harvest; separate feeding, the cows were fed roughage and concentrate separately.

**FIG 1:**
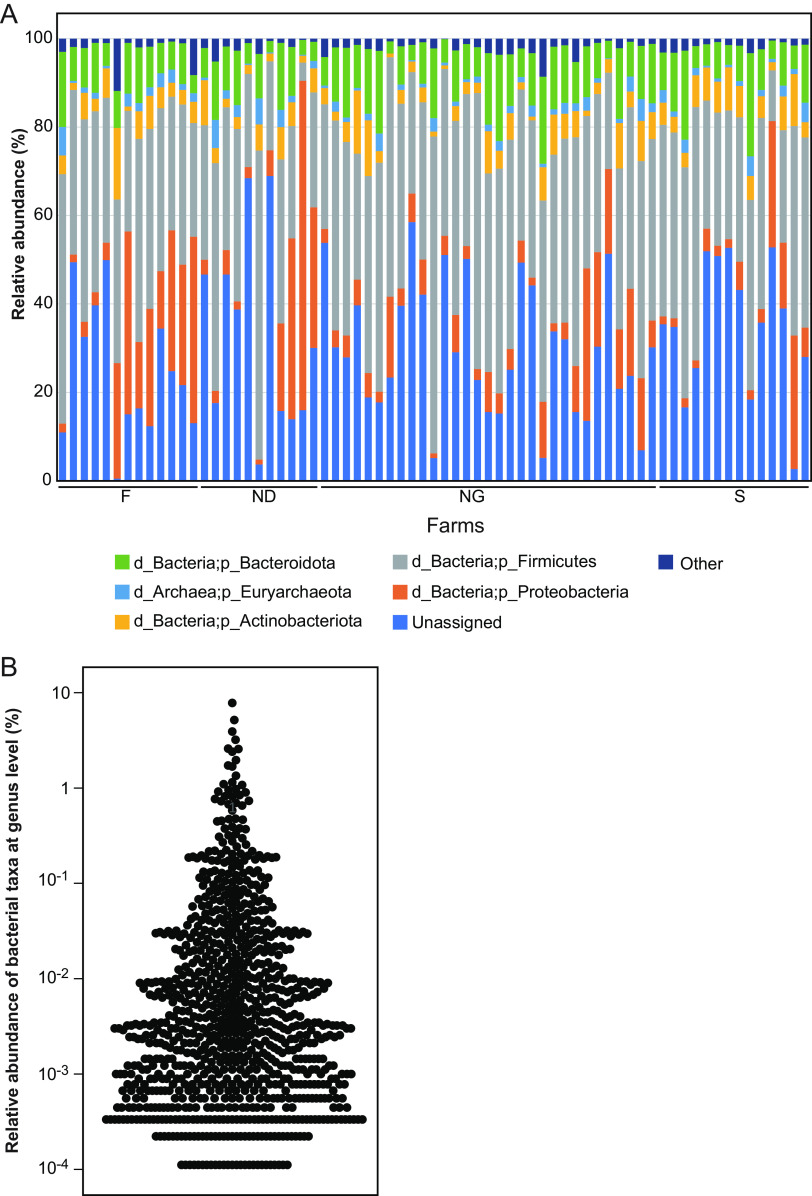
Relative abundance of uterine microbiota. (A) Taxon bar plots at the phylum level. The bacterial taxa are indicated using distinct colors, as shown on the bottom. The farms are indicated below the bar chart on the horizontal axis. The combined relative abundances totaled 100% for each cow. (B) Distribution of relative abundance at the genus level. The dots in the swarm plot represent the bacterial taxa.

We obtained 3,020,657 reads in total (mean ± standard deviation, 43,778 ± 16,666 reads/sample). Read trimming and exclusion of chimeric reads were carried out using DADA2 software and produced a total of 1,648,384 reads (23,890 ± 8,703 reads/sample). The relative abundance of the bacterial community was calculated using these data (Fig. 1A). At the phylum level, the majority of bacterial taxa were *Firmicutes*, *Proteobacteria*, *Bacteroidota*, *Actinobacteriota*, and *Euryarchaeota*, with average proportions of 38.9%, 11.6%, 10.0%, 5.0%, and 1.8%, respectively; the abundances of unassigned and other bacterial taxa were calculated to be 30.4% and 2.2%, respectively.

Moreover, the taxonomy abundance at the genus level was observed. There were 14, 77, 177, and 584 taxa at ≥1%, 1 to 0.1%, 0.1 to 0.01%, and <0.01% relative abundances, respectively (Fig. 1B). The taxa at ≥1% included *Pseudomonas* (7.8%), *Romboutsia* (5.2%), UCG-005 (family *Oscillospiraceae*) (3.9%), unassigned (family *Lachnospiraceae*) (3.2%), *Paeniclostridium* (2.6%), *Turicibacter* (2.6%), *Bacteroides* (2.4%), *Corynebacterium* (2.0%), *Clostridium sensu stricto 1* (1.7%), *Methanobrevibacter* (1.7%), *Rikenellaceae* RC9 gut group (1.3%), UCG-010 (family UCG-010) (1.1%), *Streptococcus* (1.1%), and *Lachnospiraceae* NK3A20 group (1.1%).

### Diversity analysis of uterine microbiota in the cows of four farms.

We compared the microbial diversities related to the farm, housing style, and feeding management and examined the correlation of microbial diversity with parity and the AI frequency to conception. First, alpha diversity was analyzed using Shannon and Chao1 indices. No significant differences were observed regarding the farm, housing style, and feeding management (Table 2); moreover, the pairwise analysis showed no significant differences between the groups (Fig. S2). No significant correlation of these alpha diversity indices with parity and AI frequency to conception was observed (Fig. S3 and Table S1).

**TABLE 2:** Microbial diversity analysis with respect to farm, feeding management, and housing style for the cows of all the farms

Parameter	*P* value		
	Farm	Housing style	Feed management
Alpha diversity^*a*^			
Chao1	0.697	0.469	0.553
Shannon	0.465	0.151	0.284
Beta diversity^*b*^			
Weighted UniFrac	0.198	0.196	0.09
Unweighted UniFrac	0.033	0.029	0.012

a Statistical analysis according to Kruskal-Wallis test.

b Statistical analysis using a permutational multivariate analysis of variance (PERMANOVA).

Next, beta diversity was examined by principal-coordinate analysis using unweighted and weighted UniFrac distances. Significant differences were observed using unweighted UniFrac with respect to farm, housing style, and feeding management (Table 2 and Fig. S4; *P < *0.05); however, no significant correlation was observed with parity and AI frequency to conception (Table S1; *P ≥ *0.05). The pairwise analysis in unweighted UniFrac showed a significant difference between groups with respect to housing style and feeding management (Table 3 and Fig. S4; *q *< 0.05). On the farms, the comparative groups F-NG, F-S, and NG-S exhibited a low *q* value (i.e., *q *< 0.1).

**TABLE 3:** Comparison of the unweighted UniFrac distance matrices between two groups, with respect to farm, feeding management, and housing style for the cows of all the farms

Comparison group^*a*^	Sample size	No. of permutations	Pseudo-F	*P* value	*q* value
Farm					
F vs ND	24	999	1.059941	0.287	0.4092
F vs NG	44	999	1.715226	0.020	0.0780
F vs S	27	999	1.650406	0.033	0.0780
ND vs NG	42	999	1.053892	0.341	0.4092
ND vs S	25	999	0.875121	0.637	0.6370
NG vs S	45	999	1.525050	0.039	0.0780
Feeding management					
Separate feeding vs TMR_A	38	999	1.563091	0.038	0.0460
Separate feeding vs TMR_B	44	999	1.715226	0.027	0.0460
TMR_A vs TMR_B	56	999	1.558256	0.046	0.0460
Housing style					
Free barn vs tie stall barn	69	999	1.666939	0.031	0.0310

a Statistical analysis using a pairwise PERMANOVA between groups was performed.

In contrast, no significant difference was observed using weighted UniFrac with respect to farm, housing style, and feeding management; moreover, no correlation between weighted UniFrac and parity and AI frequency to conception was observed (Table 2 and Table S1; *P ≥ *0.05). The pairwise analysis in weighted UniFrac revealed an absence of significant differences with respect to farm, housing style, and feeding management (Fig. S4 and Table S2).

Considering these results, the composition of the uterine microbiota was influenced by farm, housing style, and feeding management, not by parity or AI frequency to conception.

### Functional prediction of uterine microbial data from four farms.

We predicted the functional profile from the microbiota data using PICRUSt2 (27). The ortholog data in the predicted functional profile were analyzed by principal-coordinate analysis using the Euclidean distance. First, examining the data with respect to farm, housing style, and feeding management, significant differences were observed in all the variables (Table 4; *P < *0.05). Moreover, examining the correlation of Euclidean distance matrices with respect to parity and AI frequency to conception, the correlation was not observed on either variable (Table 5; *P > *0.05). These results suggested that the function profile varied depending on the farm, housing style, and feeding management and were almost similar to the microbiota diversity results.

**TABLE 4:** Principal-coordinate analysis of predicted functional profile with respect to farm, feeding management, and housing style for the cows of all the farms

Comparison group^*a*^	Sample size	No. of permutations	Pseudo-F	*P* value^*b*^	*q* value
Farm				**0.019**	
F vs ND	24	999	0.841423	0.432	0.4320
F vs NG	44	999	4.605098	0.004	0.024
F vs S	27	999	1.695891	0.143	0.2145
ND vs NG	42	999	4.626088	0.01	0.03
ND vs S	25	999	1.128699	0.306	0.3672
NG vs S	45	999	2.418319	0.036	0.072
Feeding management				**0.008**	
Separate feeding vs TMR_A	38	999	1.118281	0.323	0.323
Separate feeding vs TMR_B	44	999	4.605098	0.007	0.018
TMR_A vs TMR_B	56	999	3.666663	0.012	0.018
Housing style				**0.009**	
Tie stall barn	69	999	4.192042	0.006	0.006

a Statistical analysis using PERMANOVA was performed.

b *P* values in bold indicate the result of PERMANOVA in each analysis; other *P* values indicate the result of pairwise PERMANOVA between groups.

**TABLE 5:** Mantel test using Spearman’s correlation coefficient of predicted function profile, with parity and AI frequency to conception, for the cows of all the farms

Parameter	Spearman’s correlation coefficient	*P* value
Parity	−0.074521	0.128
AI frequency to conception	−0.050931	0.403

### Diversity analysis of the uterine microbiota at the NG farm in association with parity and AI frequency to conception.

Based on the above-described results showing that the farm, housing style, and feeding management influenced the uterine microbiota, we considered the possibility of a correlation of the uterine microflora with the AI frequency to conception on one farm. Because the NG farm had the highest number of study-enrolled cows among the four farms with twice to a third as many as the other three farms (Table 1), we analyzed the uterine microbiota of 31 cows kept on the NG farm. The rate of low-fertility cows on the NG farm was 39%. We reprocessed the data of sequence reads from the 31 cows using the DADA2 software and obtained a total of 678,928 reads (21,901 ± 8,334 reads/sample). We examined the correlation of microbial diversity with parity and AI frequency to conception (Table 6). First, parity was not significantly correlated with the alpha and beta diversity metrics (*P ≥ *0.05). A correlation of weighted UniFrac distance matrices with respect to AI frequency to conception was detected (*P < *0.05). Meanwhile, correlations of Chao1 and Shannon indices with AI frequency to conception were not observed (*P ≥ *0.05); those of unweighted UniFrac distance matrices with respect to AI frequency to conception were not observed (*P ≥ *0.05).

**TABLE 6:** Microbial diversity analysis of the uterine microbiota in the NG farm cows

Parameter	Parity		AI frequency to conception	
	Spearman’s rank correlation coefficient	*P* value	Spearman’s rank correlation coefficient	*P* value
Alpha diversity				
Chao1	0.0308	0.869	−0.1223	0.512
Shannon	0.0123	0.948	0.0231	0.902
Beta diversity^*a*^				
Weighted UniFrac	0.010735	0.839	0.190533	0.012
Unweighted UniFrac	−0.001651	0.970	0.009424	0.919

a The data were analyzed by the Mantel test (Spearman’s rank correlation and 999 permutations).

These results suggested that the bacterial taxa at high abundance seemed to be modified depending on the AI frequency to conception without changes in species richness at the NG farm.

### Differential abundance analysis in association with AI frequency to conception in the NG farm cows.

**FIG 2:**
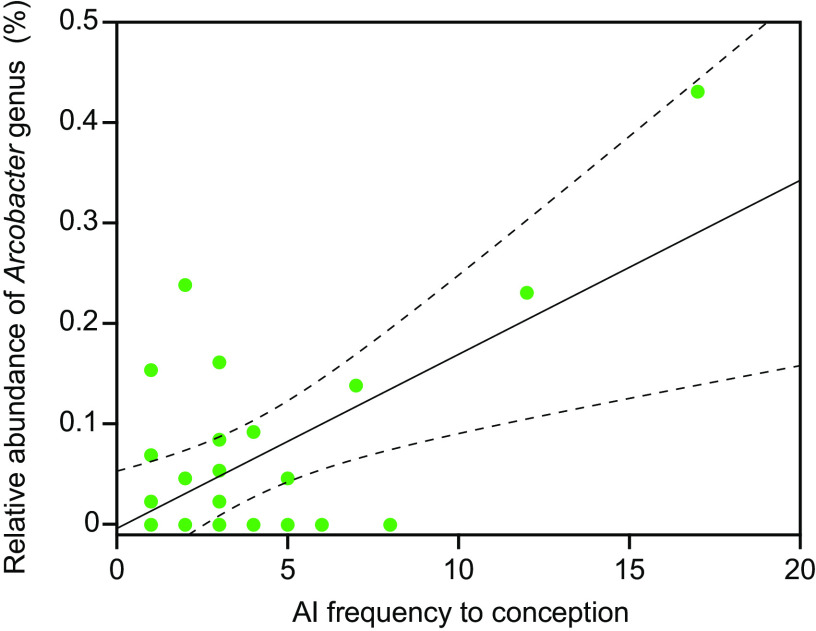
Relationship of the abundance of the *Arcobacter* genus with AI frequency to conception. The least-square curve with a 99% reliable area is indicated by a straight line with the area enclosed with dash lines. When the vertical and horizontal axes were used as *Y* and *X*, respectively, the least-square curve was calculated as *Y* = 0.0173118 × *X* – 0.003614.

We searched for the bacterial taxa at the genus level related to AI frequency to conception, using a differential abundance analysis. As a result, only the taxon of *Arcobacter* was detected to be positively associated with AI frequency to conception, while no other bacterial taxa associated with AI frequency to conception were detected (Table S3; adjusted *P < *0.05). Moreover, we further analyzed the trends of *Arcobacter* abundance in relation to AI frequency to conception by fitting the regression curve using a least-squares method (Fig. 2). The examination of the regression model using an analysis of variance identified significant differences (*P = *0.0002); 71.0% (22/31) of the cows were placed within the 99% reliable area of the model. Considering this result, the *Arcobacter* genus was considered to be positively associated with AI frequency to conception.

### Functional prediction of uterine microbial data from the NG farm.

We analyzed the functional profile of the uterine microbiota of NG farm predicted by PICRUSt2. Examining the correlation of Euclidean distance matrices with parity and AI frequency to conception, correlation with statistical significance was not observed (Table S4; *P > *0.05). Thus, the overall functional profile was not considered to be influenced by parity or AI frequency to conception. Moreover, we then searched for the functional orthologs correlated with AI frequency to conception. Three functional orthologs included K10873, K00861, and K01350, which were positively correlated with AI frequency to conception (Table S5; adjusted *P < *0.05). We then examined these functional orthologs against the Kyoto Encyclopedia of Genes and Genomes (KEGG) database, targeting only the prokaryotes. The functional orthologs K10873 and K00861 were assigned to DNA repair and recombination protein RAD52 and riboflavin kinase, respectively, while K01350 was not assigned. Thus, the function profile in the uterine microbiota appeared to be slightly modified with respect to AI frequency to conception.

### Microbial co-occurrence network patterns of the uterine microbiota associated with low fertility in the NG farm cows.

**FIG 3:**
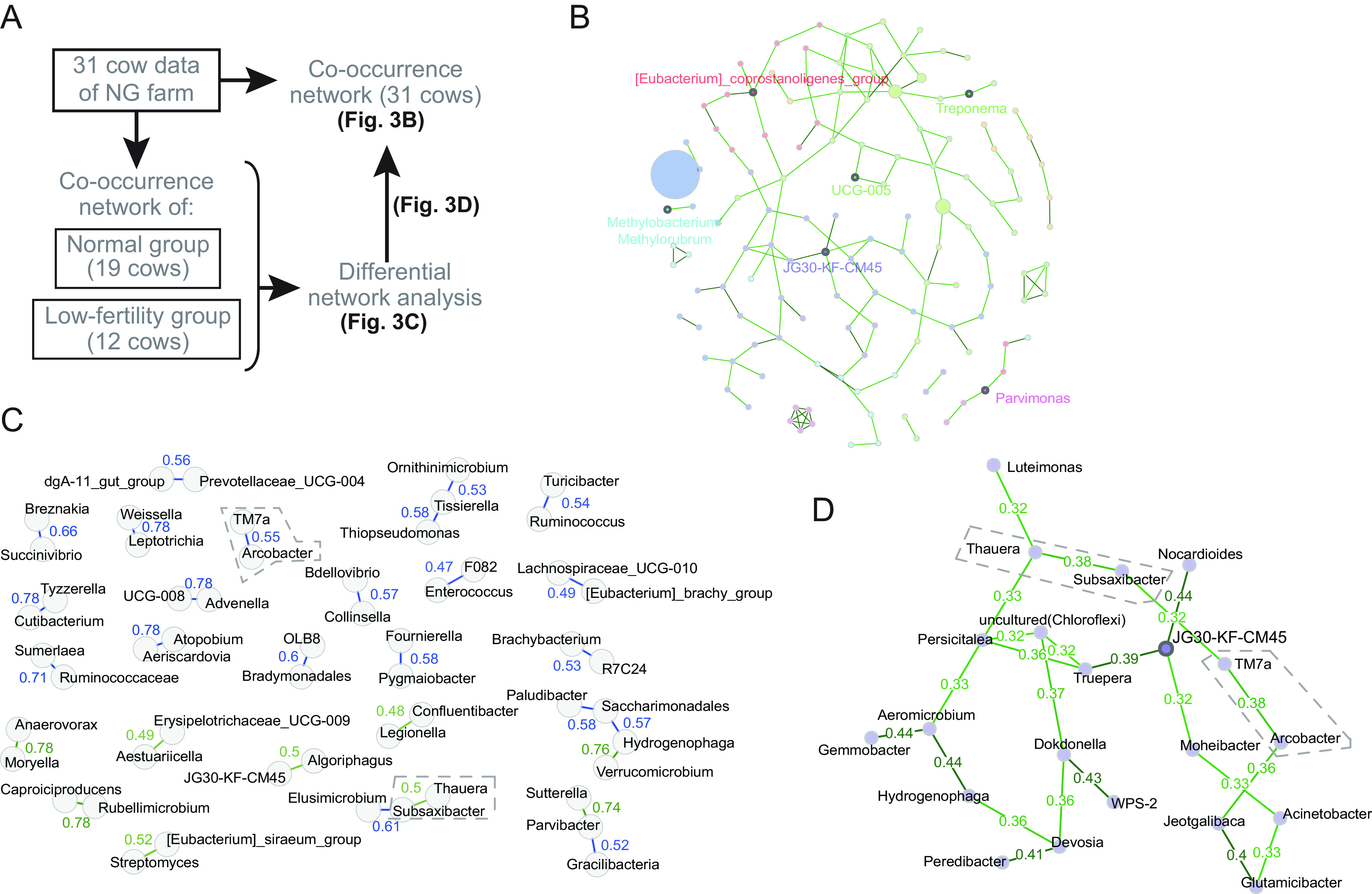
Assumption of bacterial associations by microbial co-occurrence network analysis in the NG farm. (A) Analysis procedure to assume the bacterial associations related to fertility. (B) Co-occurrence network of the uterus microbiota of all the cows in the farm. The node size represents the abundance of bacterial taxa. The numbers adjacent to the edges are the edge weights. Bold points with a name in the network represent the hubs calculated from the centralities. In the network, based on the network centralities, *Eubacterium_coprostanoligene* (order *Oscillospirales*), *Methylobacterium-Methylorubrum* (family *Beijerinckiaceae*), *Parvimonas* (order *Peptostreptococcales-Tissierellales*), JG30-KF-CM45 (order *Thermomicrobiales*), Treponema (family *Spirochaetaceae*), and UCG-005 (family *Oscillospiraceae*) were assumed to be hub bacteria. (C) Normal-group-preponderance and low-fertility-group-preponderance associations detected in a differential network. Thirty edges with the highest differences were extracted from the differential network analysis. Green and blue lines are normal-group-associated and low-fertility-group-associated edges, respectively. The edge weights are shown in numbers adjacent to the edges. (D) A cluster of co-occurrence network of the uterus microbiota, containing JG30-KF-CM45 as a hub. The cluster, which is a part of the co-occurrence network of the NG farm cows, contained the low-fertility-group-preponderance association of *Arcobacter*-TM7 and the normal-group-preponderance association of *Thauera*-*Subsaxibacter*, which are enclosed with dashed lines in panels C and D.

Understanding the microbial co-occurrence network analysis can provide insights into the robustness of ecological systems (28). The microbial co-occurrence network helps decipher complex microbial association patterns, and differential network analysis deciphers the bacterial associations differentially associated between two groups (28). In this study, the cows that conceived within three AIs were categorized as being normal, whereas the cows that underwent three or more consecutive AIs without conception were categorized as having low fertility. Of 31 cows on the NG farm, 19 and 12 were categorized into normal (≤3 AIs) and low-fertility (≥4 AIs) groups, respectively. We estimated the bacterial associations with normal and low fertility by a combination method of co-occurrence network analysis of all cows at the NG farm with differential network analysis of the two groups (Fig. 3A).

First, the microbial association estimation at the genus level was made by the conditional dependence method using the microbiota data of NG farm, and the co-occurrence network was constructed (Fig. 3B and Fig. S5). Next, we estimated the co-occurrence patterns of the normal and low-fertility groups by the method described above, and the differential network was produced from the comparison of co-occurrence patterns using a nonparametric method (Fig. S6). Thirty edges with the highest weights were selected from the differential network (Fig. 3C), including 21 low-fertility-group-preponderance and 9 normal-group-preponderance associations. Moreover, these 30 highly weighted associations in the differential network were searched in the co-occurrence network derived from the NG farm cows (Fig. S5). As a result, the low-fertility-group-preponderance association of *Arcobacter*-TM7 and the normal-group-preponderance association of *Thauera*-*Subsaxibacter* were found in the cluster containing JG30-KF-CM45 as a hub (Fig. 3D); the other associations were not found in the network. The relative abundances of *Arcobacter*, TM7, *Thauera*, and *Subsaxibacter* on the NG farm were, on average, 0.031%, 0.036%, 0.048%, and 0.021%, respectively.

## DISCUSSION

The present study focused on the uterine microbiota of dairy cows that had passed the VWP to examine the relevancy to fertility. First, the microbiota of cows housed on four farms was described at the phylum and genus levels. The uterine microbiota was found to vary depending on the farm management practices such as housing style and feeding management of cows were overall similar to those in the other study at the phylum level; however, they were not similar at the genus level, and the farm management practices, such as housing style and feeding management, were found to influence the uterine microbiota among farms. Next, the study focusing on the NG farm revealed that the diversity of the uterine microbiota was correlated with the AI frequency to conception. Furthermore, a study in correlation with AI frequency to conception revealed the partial modification of the predicted function profile and relation of a single bacterial taxon *Arcobacter*. The bacterial associations related to fertility were estimated.

First, the majority of bacterial taxa obtained in the study were, in order of abundance of the phyla, *Firmicutes*, *Proteobacteria*, *Bacteroidota*, *Actinobacteriota*, and *Euryarchaeota*. With reference to the several studies using a uterine lavage approach at a similar postpartum period, these phyla were also found to be predominant, indicating that they are the major bacteria present in the uterus of cows, although the order of abundance differs (22, 29). This suggested that the bacterial taxa obtained from the uterus of cows were overall similar to the other study at the phylum level. On the contrary, the difference was also observed in abundance at the genus level (29). The genital microbiota of cows is recognized to vary depending on the examination method, the condition of cows, and the environment in which the cows are placed (24, 30, 31). Thus, this difference in uterine microbiota may be attributed to the sampling method as well as factors such as the breed of cow, farm management practices, and regional characteristics.

Second, the farm management practices, such as housing style and feeding management, were found to influence the microbial diversity, suggesting the importance of confirming the variation in the uterine microbiota among farms. In addition, the farm management practices appeared to be reflected in the functional profile. The variety of housing styles on dairy farms (e.g., tie stall barn, free stall barn, free barn, etc.), as well as the bedding materials (e.g., barley straw, woodchips, sand, etc.), are reported to contribute to the establishment of unique uterine and fecal microbiotas on individual farms (31, 32). For feeding management, different food components, such as fiber and starch, are regarded as forming different gut microbiota (33, 34). The gut microbiota regulates the estrogen level in the blood mediated by secretion of β-glucuronidase (35), and changes in estrogen level may influence the uterine microbiota in the cows. In addition, the gut microbiota may translocate hematogenously from the gut to the uterus and may also be the cause of a possible change in the uterine microbiota (36). Thus, the formation of the uterine microbiota is dependent on farm management practices.

Third, because farm management practices influence the formation of the uterine microbiota, we believed that the analysis of one farm could elucidate a farm-specific relationship between the uterine microflora and fertility. Analysis of the uterine microbiota of cows housed on the NG farm showed that the diversity of the uterine microbiota correlated with AI frequency to conception. Subsequent differential abundance analysis of the uterine microbiota showed that the *Arcobacter* genus positively correlated with AI frequency to conception. *Arcobacter* has been reported to be associated with ruminant abortions, which suggested the possibility of reducing fertility (37). The pathogenesis of *Arcobacter* is referred as the adhesion, invasion, and cytotoxic potential of this bacterium in *in vitro* settings (38). However, the relative abundance of *Arcobacter* was not high, averaging 0.031% on the NG farm, and *Arcobacter* was not considered to be a major bacterial taxon group. In addition, because the detected pathways were limited within the functional profile analysis, the overall effects on the uterine environment in relation to fertility were difficult to estimate. The above-described results suggested that it was unlikely to have an immediately direct impact on uterine environments in low-fertility cows on the NG farm.

Finally, a co-occurrence network analysis was performed on the cows of NG farm. The low-fertility-group-preponderance association of *Arcobacter-TM7* and the normal-group-preponderance association of *Thauera*-*Subsaxibacter* were detected from the cluster containing JG30-KF-CM45 as a hub in the co-occurrence network of the NG farm. These two associations found in this study can be keystones with respect to the fertility on the NG farm. *Arcobacter* has been discussed to co-occur with other bacteria, to affect the maternal maintenance of pregnancy (37), and, as discussed above, to be one of the possible etiologic infectious abortion agents in ruminants. TM7, which is symbiotic bacterium recalcitrant to the conventional culture method, has been reported to be associated with periodontitis (39). On the other hand, although the function of *Subsaxibacter* is scant to our knowledge, *Thauera* is reported to degrade excessive amounts of testosterone (40), potentially having a positive effect on fertility, as testosterone present in the endometrium has a detrimental effect on reproductive function (41). Thus, the uterine microbiota in cows on the NG farm is likely to be balanced in relation to fertility.

Based on this study, two points can be discussed for the future application of the uterine microbiota in cows: the possible functions in uterus and the biomarker for low fertility. First, recent studies have speculated that the uterine microbiota contributes to the regulation of endometrial physiology and reproductive function, thereby affecting fertility, although direct evidence is still lacking (42). Because subtle changes in the uterine microbiota and function profile were observed, we considered that a significant alternation in endometrial functions is not present on the NG farm. However, more obvious changes in uterine function could be observed on other farms, as the present study showed farm-to-farm variation in the formation of the uterine microbiota. Thus, multiple studies on individual farms examining the uterine microbiota associated with fertility are required to estimate direct effects on the endometrium. Second, it is of concern whether the *Arcobacter* abundance can be a biomarker for low fertility in general, because *Arcobacter* was detected to be associated with low fertility in both differential abundance and co-occurrence network analyses on the NG farm. The differential abundance analysis of uterine microbiota on the other three farms did not detect positive associations of *Arcobacter* taxon with AI frequency to conception, either by statistical significance or by low adjusted *P* values (data not shown). The factors influencing low fertility and the featured bacteria in the uterine microbiota may be different from farm to farm. To discover the common microbial biomarker for low fertility, studies with a large number of cows housed on various farms are required.

In human practice, the uterine microbiota is becoming generally utilized as a biomarker for a poor reproductive prognosis. In addition, treatment to normalize the uterine microbiota mediated by gut immunity, such as oral administration of prebiotics, has been attempted based on the outcome of uterine microbiota testing (43). The present study provided new insights into the relationship of the uterine microbiota with fertility in dairy cows, suggesting that uterine microbiota testing may be applicable in cows. We hope that uterine microbiota data will be accumulated from a variety of cows, leading to the establishment of the examination system and its application to solve problems associated with low fertility.

### Conclusion.

This metataxonomic analysis using bovine endometrium illustrated the potential associations of uterus microbiota with AI frequency to conception, which deciphers the two following points. (i) Because the uterine microbiota varied on a farm-by-farm basis, the causative factors for low fertility with respect to the uterine microbiota may differ. (ii) The small change in the uterine microbiota, and the cooperation between bacteria, may be related to unexplained low fertility. It is hoped that the accumulation of uterine microbiota data will help to develop an examination system for unexplained low fertility in cows and contribute to improvement in reproductive management.

## MATERIALS AND METHODS

### Animals.

Ninety-eight Holstein-Friesian cows (4.3 ± 1.0 years old; 2.8 ± 0.9 parity; 3.0 ± 0.1 body condition score; 71.4 ± 7.2 days postpartum) were enrolled in the study between October 2018 and February 2021. The cows were kept on four commercial dairy farms (F, ND, NG, and S) in Hokkaido, Japan. Cows on these farms were fed under different feeding managements: the F farm fed cows by a separate feeding, i.e., a method to supply feeds separately, and the ND, NG, and S farms fed cows by a total mixed ration (TMR), i.e., a method to supply feeds in a mixture. Among the farms that fed TMR, the NG farm fed TMR prepared by the farm owner (TMR_A) and the ND and S farms fed purchased TMR (TMR_B). Diets were formulated in each farm using the National Research Council (2001) model or Cornell-Penn-Miner dairy model (44) to satisfy nutritional requirements of 30 to 40 kg/day of milk production (Table S6). The F, ND, and S farms housed cows in a tie stall barn, and the NG farm housed cows in a free barn.

The inclusion criteria for cows were as follows: a parity of 2 to 5, a body condition score of 2.75 to 3.25, no history of treatment for systemic disease within 30 days, no signs of lameness (45, 46), and a clinically healthy genital tract. The health status of the genital tract was confirmed by vaginoscopy and ultrasonography. Vaginoscopy assessed the vaginal discharge score as a diagnostic indicator of clinical endometritis using a scale of 0 to 5: 0, no discharge, 1, clear mucus, 2, mucus with flecks of pus, 3, mucopurulent discharge, 4, purulent discharge, and 5, foul-smelling discharge (47). Ultrasonography evaluated abnormalities based on the diameter of the images depicted: in the uterus, a uterine luminal fluid greater than 2 mm (48), and in the ovaries, an ovarian cyst filled with anechoic structures greater than 25 mm (49). Cows with a vaginal discharge score of <2 and no abnormalities by ultrasonography were included in the study. During the monitoring period, the cows that exhibited systemic symptomatic disease, lameness, or genital tract abnormalities were excluded from the study. No evaluation was made in this study regarding the genetic ability for the reproductive performance of the cows (50, 51).

### Experimental design.

The biopsy samples were collected between 60 and 85 days postpartum during the diestrus phase, which was determined by the size of the corpus luteum (>20 mm in diameter) using ultrasonography (49, 52, 53) and the absence of estrus signs based on behavioral observation, vaginoscopic examination, and uterine palpation (54). Once the samples were collected, AI was performed after estrus detection and ovulation was confirmed on the day after AI. To avoid the embryonic lethality possibly caused by the Holstein-Friesian lethal haplotype, the selection of semen and AI was conducted after checking for the carriage of the known haplotype in the bulls (55, 56). AIs were repeated until pregnancy. Pregnancy was diagnosed ca. 60 days after AI using ultrasonography (Tringa Linear, Esaote Pie Medical, Genoa, Italy).

### Biopsy sampling.

Endometrial tissues were collected using a biopsy instrument (Fujihira Kogyo Co., Ltd., Japan) under caudal epidural anesthesia with 5 mL of 2% procaine hydrochloride, as described previously (57). The endometrial tissue at each bifurcation of the right and left uterine horns was collected by pressing the sample against the hole in the outer cylinder and hollowing it out with the inner cylinder. The samples were cut into small pieces immediately at the farm and placed in 2 mL of phosphate buffer at 4°C. The solution containing the sample was stirred and temporarily stored at −20°C. The biopsy samples were stored at −80°C at the laboratory until use.

### Extraction and examination of DNA.

Biopsy samples suspended in phosphate-buffered saline (PBS) were directly subjected to DNA extraction using an ISOSPIN fecal DNA kit (Nippon Gene, Tokyo, Japan) according to the manufacturer’s instructions. The DNA solutions were examined using a spectrophotometer (NanoDrop 1000; Thermo Scientific, Wilmington, DE, USA) to measure the DNA quality and amount. To determine the study enrollment, the presence and quality of bacterial DNA were then examined by PCR targeting the 16S rRNA gene, as described previously (58).

### Sequencing.

The DNAs were subjected to 16S rRNA gene amplicon sequencing. The V3-V4 region of the 16S rRNA gene that was amplified by PCR with forward primer 341F (5′-CCTACGGGNGGCWGCAG-3′) and reverse primer 785R (5′-GACTACHVGGGTATCTAATCC-3′) was analyzed using Illumina MiSeq with a MiSeq reagent kit v3 (600 cycles; Illumina, San Diego, CA, USA), as described previously (59).

### Data processing and taxonomy assignment.

The sequence data were processed using the Quantitative Insights into Microbial Ecology 2 (QIIME2) pipeline v. 2021.11.0 (60). The DADA2 software package v2021.8.0 incorporated into QIIME 2 was used to correct the amplicon sequence errors and to construct an amplicon sequence variant table. The amplicon sequence variant table was rarefied. Microbial taxonomy was assigned using a pretrained taxonomical classifier available on the QIIME2 webpage (a naive Bayes classifier trained on the Silva 138 99% operational taxonomic unit [OTU] full-length sequences; https://github.com/qiime2/docs/blob/master/source/data-resources.rst; accessed 1 April 2022).

### Analysis of microbial diversity.

Metrics of alpha diversity, including Faith’s phylogenetic diversity (Faith-PD), the Chao1 index (Chao1), and Shannon’s index (Shannon), and those of beta diversity, including unweighted and weighted UniFrac, were calculated using the QIIME2 pipeline (60).

The data were analyzed using statistics implemented in QIIME2. The data were examined with respect to categorical and numerical variables by the Kruskal-Wallis test and Spearman’s correlation coefficient, respectively. In the principal-coordinate analysis, the distant metrics were analyzed with categorical and numeric variables by permutational multivariate analysis of variance (PERMANOVA) and the Mantel test using Spearman’s correlation coefficient, respectively.

### Analysis of predicted functional profile.

The microbial profile was predicted using PICRUSt2 plugin q2-picrust2 2021.11_0, which was installed in QIIME2 v2021.8.0 (27, 60, 61). The Euclidian distance was obtained from the predicted functional profile data of KEGG orthologs (i.e., KO), using the QIIME2 pipeline. In the principal coordinate analysis, the distant metrics were analyzed with categorical and numeric variables using Spearman’s correlation coefficient by PERMANOVA and the Mantel test, respectively. Moreover, the KEGG ortholog data were analyzed by differential abundance analysis described below. The KEGG ortholog data were examined in the KEGG database (https://www.genome.jp/kegg/; accessed 11 January 2023) (62).

### Differential abundance analysis.

The differential abundance analysis was done using the software package DAtest v2.8.0 in R statistical software, v. 4.1.3 (63). The statistical models were examined with the data using the “testDA” function with 1,000 repeats (Tables S7 and S8). The microbiota abundance data and the predicted function profile data were analyzed using the quasi-Poisson generalized linear model and Pearson correlation coefficient, respectively, which were implemented in the DAtest package. The statistical significance was set as an adjusted *P* value of <0.05.

### Microbial co-occurrence network analysis.

Microbial associations were estimated from the microbiota data at the genus level by the SPRING model using 150 edges with the highest variance (64). The co-occurrence network was constructed with the default setting with the removal of singletons. The cluster was inferred using the Louvain method. In the differential network analysis, the associations in the networks were compared via a permutation test with 1,000 replicates, and the differential associations with an alpha of 0.05 were selected. All the co-occurrence network analyses and their visualization were performed using the NetCoMi package v. 1.0.2 in R software v. 4.0.2 (65). Finally, the associations in the co-occurrence network were compared with the associations with the highest weights in the differential network, and the associations that were seen in both networks were highlighted as low-fertility-group-preponderance and normal-group-preponderance associations.

### Visualization for figures.

The figure visualization was done using JMP Pro 16.2.0 software (SAS Institute, Cary, NC, USA) or R software v. 4.0.2 (65), unless otherwise stated.

### Ethics approval and consent to participate.

The farms included in the study expressed willingness to cooperate and had data available for reproduction and disease management. The care and handling of the animals were in accordance with Azabu University animal experiment guidelines. All experiments were reviewed and approved by the Ethics Committee of Azabu University (approval number 210720-5).

### Data availability.

Raw sequence data were deposited in the DNA Data Bank of Japan (DDBJ) sequence read archive (DRA014763) under BioProject no. PRJDB14269.
