# Supplementary material for: Metataxonomic Analysis of the Uterine Microbiota Associated with Low Fertility in Dairy Cows Using Endometrial Tissues Prior to First Artificial Insemination
Source: Microbiol Spectr. 2023 Apr 26;11(3):e04764-22. doi: 10.1128/spectrum.04764-22 (PMC10269553; doi:10.1128/spectrum.04764-22)
Supplement: Supplemental file 1 — Fig. S1 to S6 and Tables S1 to S8. Download spectrum.04764-22-s0001.pdf, PDF file, 1.1 MB [file spectrum.04764-22-s0001.pdf]

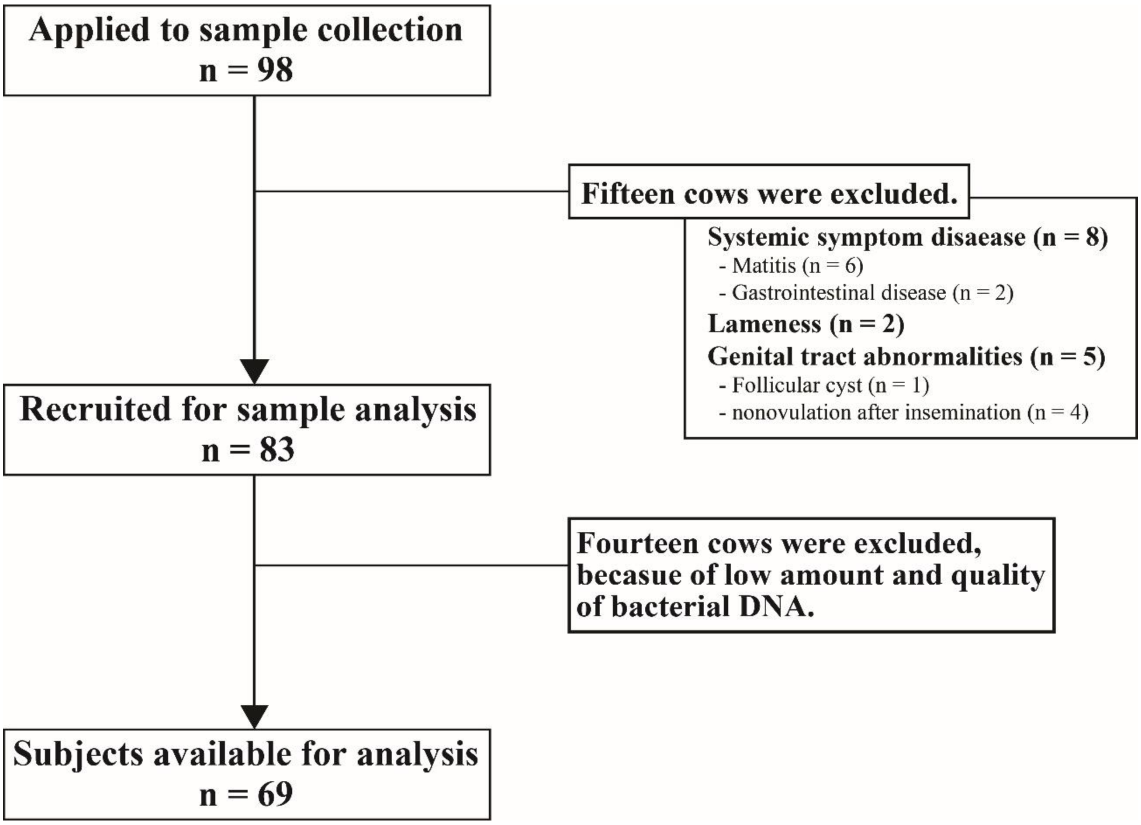

FIG. S1 Flow chart of the sample selection procedure. Fifteen cows were initially excluded from 98 cows, because of the presence of systemic symptomatic disease, lameness, or genital tract abnormalities. Among the 83 cows, 14 cows were excluded because of a low amount and quality of bacterial DNA. Although some cows were culled for failure to conceive during monitoring, these animals were included in this study because they had a generally greater AI frequency to conception (*i.e.*, ≥ 4 AIs). Sixty-nine cows were enrolled in this study.

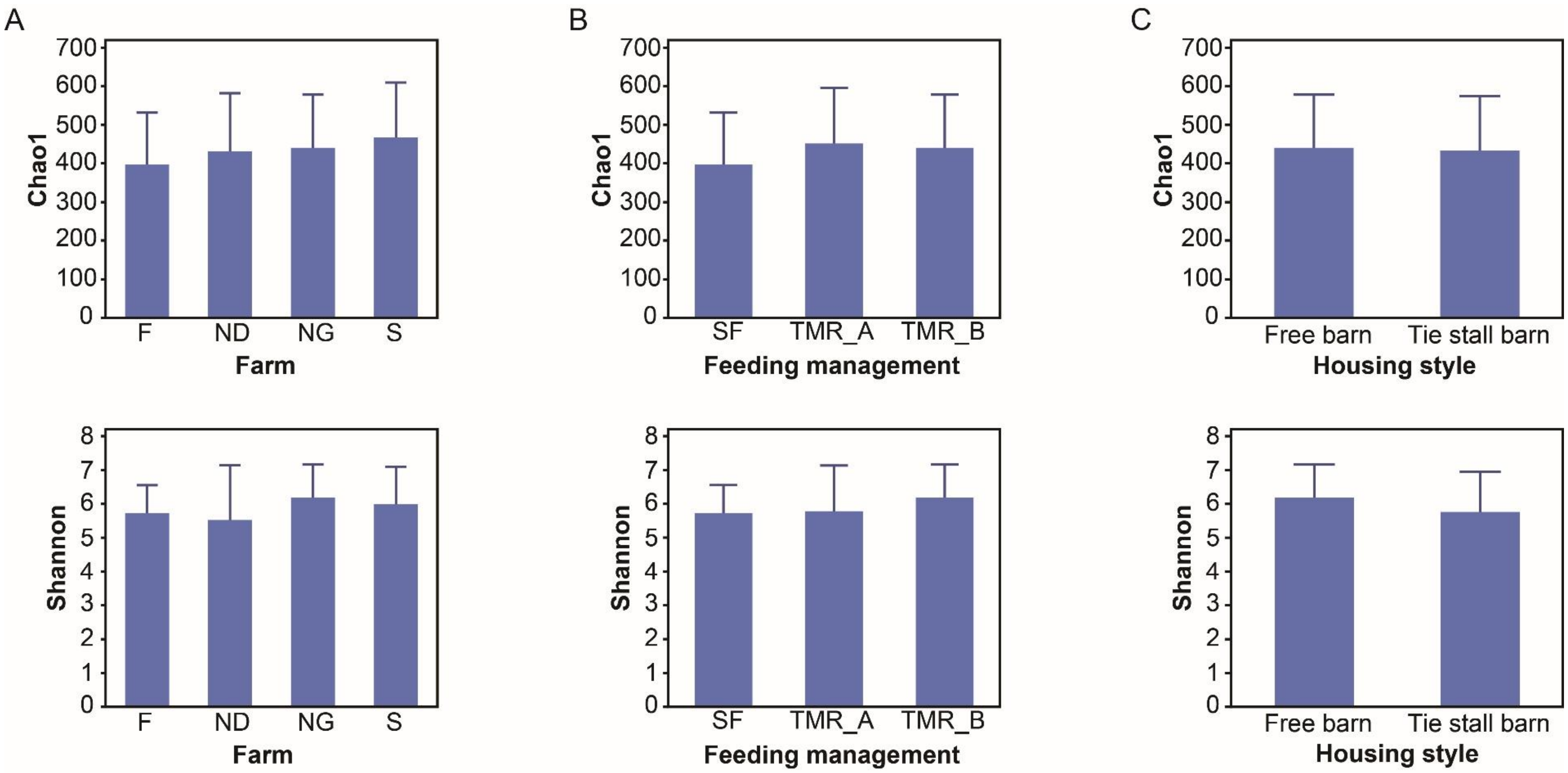

FIG. S2 Analysis of the alpha diversity metrics, Chao1 and Shannon, according to (A) farm, (B) feeding management, and (C) housing style. Kruskal–Wallis test was performed. No statistically significant differences were observed among the groups.

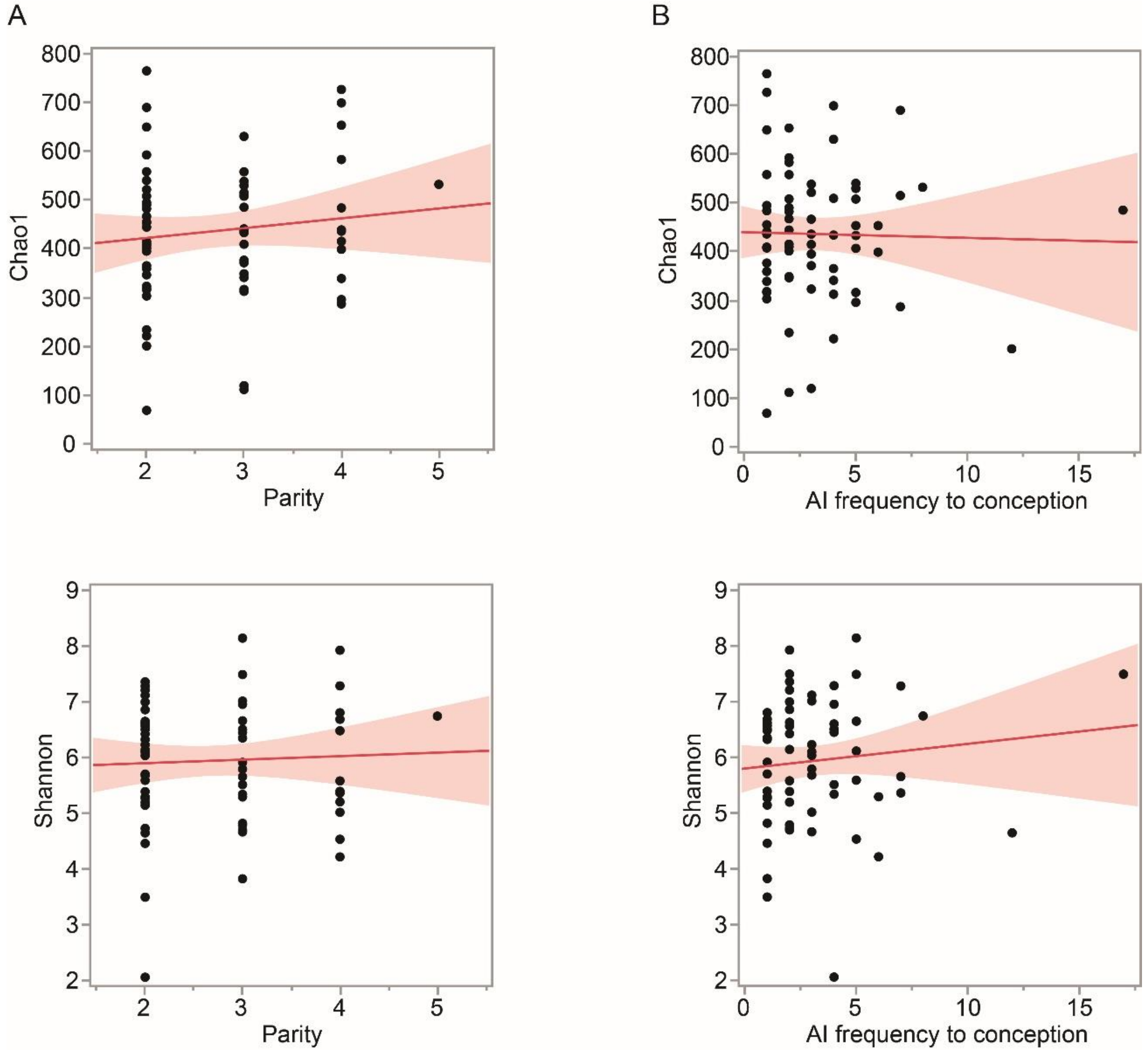

FIG. S3 Scatter plots of the alpha diversity metrics, Chao1 and Shannon, according to (A) parity, and (B) AI frequency to conception. The linear lines are fitted to the plot graph. The red areas are the reliability of the fitted lines at 99%.

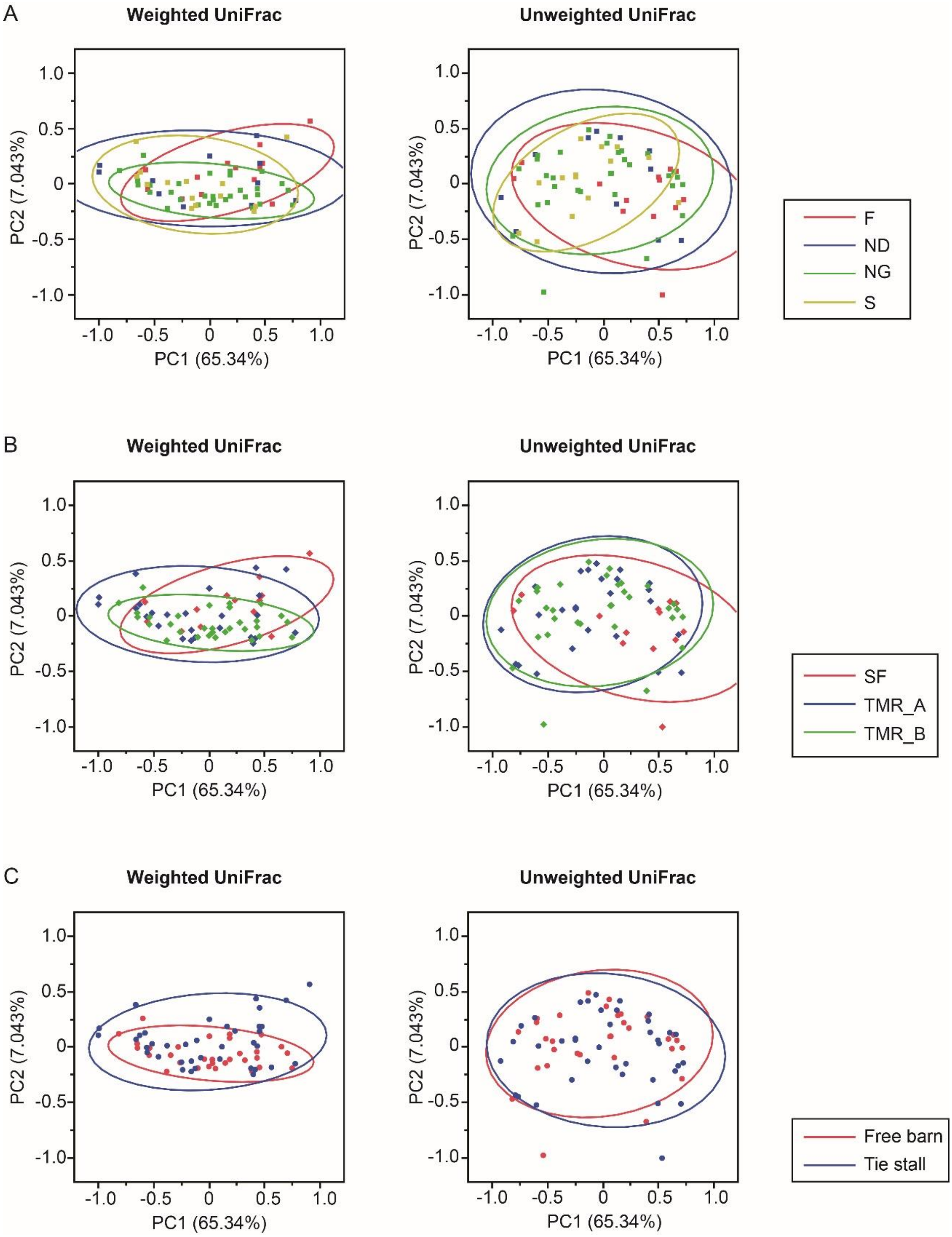

FIG. S4 Principal coordinate analysis of weighted and unweighted UniFrac. (A) Farm. (B) Feeding management. (C) Housing style. The ellipses of probability (90%) are shown on the graphs. In the rightmost boxes, the groups are indicated using different colors.

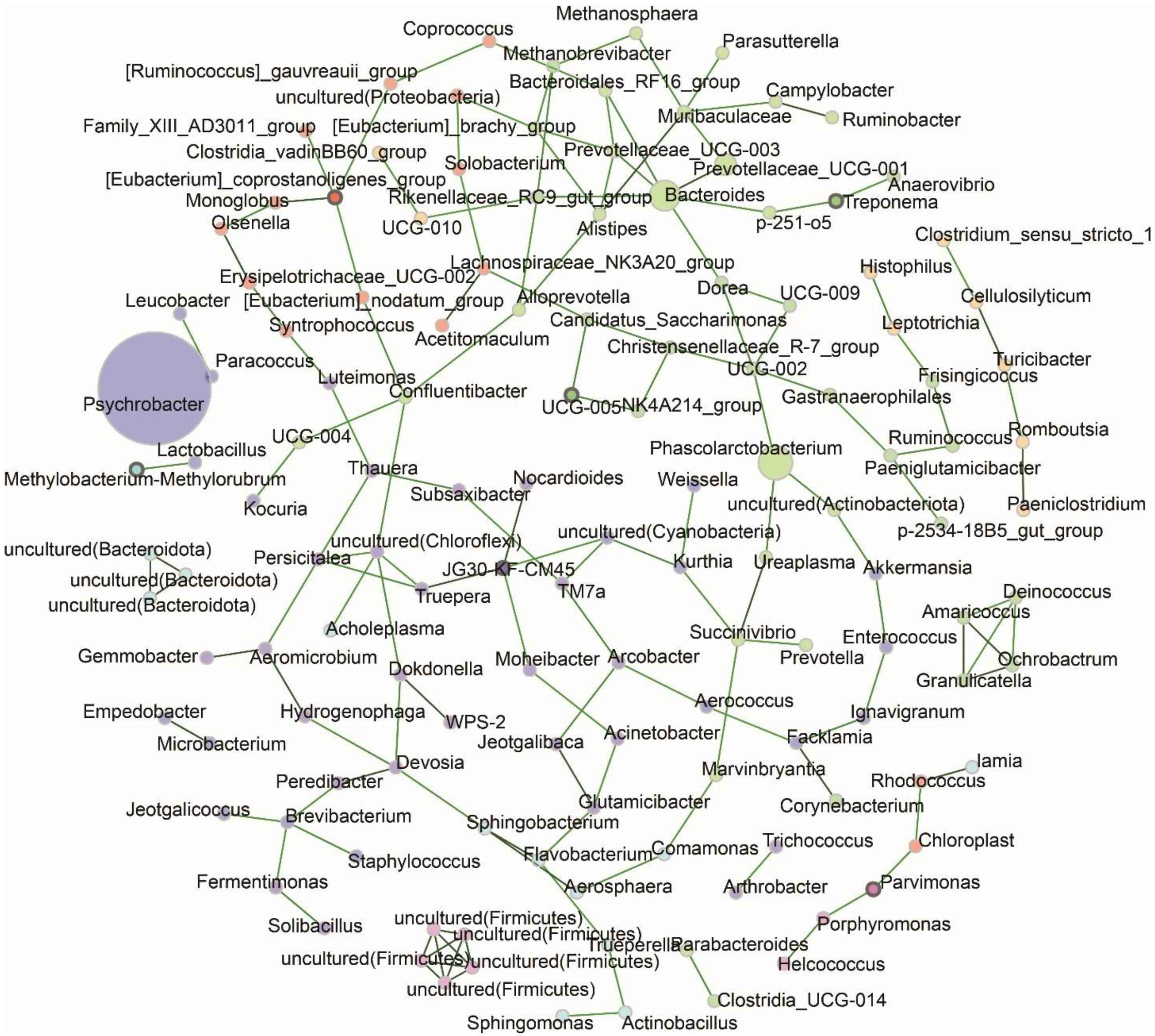

FIG. S5 Microbial co-occurrence network of the uterine microbiota in the NG-farm cows. The cluster was inferred using the Louvain method. The network was shown, and 150 edges with the highest variances were selected and singletons were removed. Different node colors represent the clusters. The name of bacterial taxa at the genus level is shown.

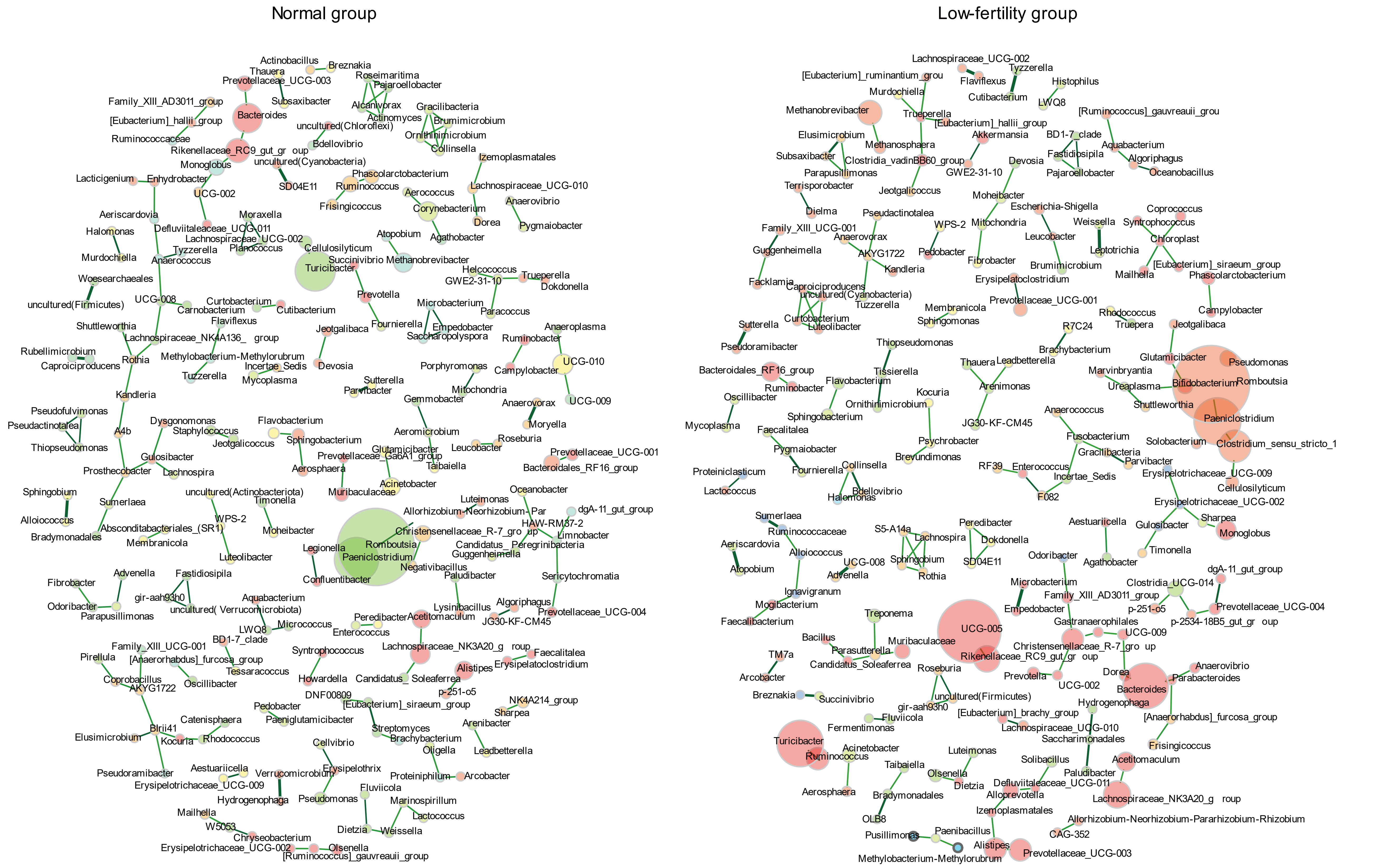

FIG. S6 Microbial co-occurrence network of the uterine microbiota of the normal and low-fertility groups. The network was shown, and 150 edges with the highest weights were selected and singletons were removed. Different node colors represent the clusters. The line thickness represents the association weight. The networks of the normal and low-fertility groups are shown on the left and right, respectively.

TABLE S1 Correlation analysis of alpha and beta diversity regarding parity and AI frequency to conception in the cows of all the farms

	**Parity**		**AI frequency to conception**	
	**Spearman's rank correlation coefficient (ρ)**	***P* value**	**Spearman's rank correlation coefficient (ρ)**	***P* value**
Alpha diversity				
Chao1	0.0723	0.555	–0.0044	0.972
Shannon	0.0174	0.887	0.1317	0.287
Beta diversity[1]				
Weighted UniFrac	–0.0082	0.828	0.0508	0.256
Unweighted UniFrac	–0.0182	0.689	0.0109	0.824

The data were analyzed by the Mantel test (Spearman's rank correlation and 999 permutations).

TABLE S2 Comparison of weighted UniFrac distances matrices among two groups, with respect to farm, feeding management, and housing style, in the cows of all the farms

**Comparison groups**			**Sample size**	**Permutations**	**pseudo-F**	***P* value**	***q* value**
Farm							
F	vs.	ND	24	999	1.187222	0.274	0.3288
		NG	44	999	2.00908	0.125	0.3288
		S	27	999	2.325901	0.101	0.3288
ND	vs.	NG	42	999	1.398976	0.231	0.3288
		S	25	999	0.450809	0.666	0.666
NG	vs.	S	45	999	1.508489	0.196	0.3288
Feeding management							
Separate feeding	vs.	TMR_A	38	999	2.088741	0.132	0.144
		TMR_B	44	999	2.00908	0.116	0.144
TMR_A	vs.	TMR_B	56	999	1.831873	0.144	0.144
Housing style							
Free barn	vs.	Tie stall barn	69	999	1.528385	0.196	0.196

Statistical analysis using a permutational multivariate analysis of variance (PERMANOVA).

TABLE S3 Differential abundance analysis of uterine microbiota of NG farm with AI frequency to conception using a quasi-Poisson generalized linear model

**Bacterial taxa**[1]	**(Intercept)**	**Predictor**	***P* value**	**log$_2$FC**	**Adjusted *P* value**
d_Bacteria; p_Campilobacterota; c_Campylobacteria; o_Campylobacterales; f_Arcobacteraceae; g_Arcobacter	1.17	0.17	1.5×10^{-4}	0.2428	0.03
d_Bacteria; p_Bacteroidota; c_Bacteroidia; o_Flavobacteriales; f_Weeksellaceae; g_Chryseobacterium	1.35	0.16	9.2×10^{-3}	0.2367	0.58
d_Bacteria; p_Bacteroidota; c_Bacteroidia; o_Chitinophagales; f_Chitinophagaceae; g_Taibaiella	0.55	0.14	8.6×10^{-3}	0.2040	0.58
d_Bacteria; p_Verrucomicrobiota; c_Verrucomicrobiae; o_Verrucomicrobiales; f_Akkermansiaceae; g_Akkermansia	3.09	0.08	0.01	0.1205	0.58
d_Bacteria; p_Proteobacteria; c_Alphaproteobacteria; o_Rhodobacterales; f_Rhodobacteraceae; g_Paracoccus	2.05	0.14	0.02	0.2059	0.66
d_Bacteria; p_Firmicutes; c_Clostridia; o_Oscillospirales; f_Ruminococcaceae; g_Ruminococcus	4.65	0.06	0.03	0.0843	0.83
d_Bacteria; p_Firmicutes; c_Clostridia; o_Oscillospirales; f_Ruminococcaceae; g_Incertae_Sedis	0.70	0.11	0.03	0.1599	0.83

[1], Only the bacteria taxa with $P < 0.05$ are shown.

TABLE S4 Mantel test using Spearman's correlation coefficient of predicted function profile, with parity and AI frequency to conception in the NG-farm cows

	**Spearman's correlation coefficient (ρ)**	***P* value**
**Parity**	–0.071013	0.259
**AI frequency to conception**	0.002433	0.983

TABLE S5 Differential abundance analysis of predicted functional ortholog data of NG farm with AI frequency to conception using Pearson correlation coefficient

KEGG ortholog[1]	*P* value	Adjusted *P* value	Correlation coefficient
K10873	4.209E-06	0.018	0.724
K00861	9.877E-06	0.018	0.704
K01350	9.877E-06	0.018	0.704
K15897	2.007E-04	0.290	0.620
K06931	4.910E-04	0.590	0.589
K15491	0.001	0.999	0.550
K12211	0.001	0.999	0.548
K01732	0.002	0.999	0.544
K17398	0.002	0.999	0.538
K12223	0.002	0.999	0.538
K19519	0.003	0.999	0.515
K13873	0.003	0.999	0.514
K15765	0.003	0.999	0.514
K18430	0.003	0.999	0.512
K16928	0.003	0.999	0.509
K14446	0.006	0.999	0.485
K13652	0.006	0.999	0.481
K12506	0.006	0.999	0.480
K12204	0.007	0.999	0.475
K05988	0.007	0.999	0.474
K12995	0.007	0.999	0.473
K14195	0.007	0.999	0.473
K18782	0.008	0.999	0.470
K12061	0.009	0.999	0.463
K11048	0.009	0.999	0.462
K05994	0.009	0.999	0.460
K11052	0.010	0.999	0.457
K14192	0.010	0.999	0.457
K11039	0.010	0.999	0.457
K18923	0.010	0.999	0.457
K01227	0.010	0.999	0.455

[1], Only the KEGG orthologs with $P \leq 0.01$ are shown.

TABLE S6 Ingredients and nutritional composition of basal diet fed at each farm throughout the study period

	Farm		
	**F**	**ND and S[1]**	**NG**
Dry matter, kg	25.0 ± 0.3	24.7 ± 0.7	23.6 ± 2.4
Ingredients, % of dry matter (average ± standard deviation)			
Corn silage	30.2 ± 3.8	36.9 ± 4.56	22.4 ± 3.6
Grass silage	–	22.2 ± 4.1	22.9 ± 4.6
Grass hay	15.1 ± 2.4	4.0 ± 0.9	–
Concentrates	36.6 ± 2.4	29.5 ± 3.3	40.9 ± 2.0
Beet pulp	11.1 ± 1.5	4.2 ± 1.5	7.8 ± 3.8
Bypass protein supplement	–	3.2 ± 0.6	–
Soybean meal	4.5 ± 0.5	–	4.7 ± 1.4
Sodium bicarbonate	–	–	0.3 ± 0.2
Calcium carbohydrates	–	–	1.0 ± 0.4
Vitamin and mineral supplement	1.6	–	–
Nutrient composition, % of dry matter (average ± standard deviation)			
Crude protein	15.6 ± 0.3	15.1 ± 0.4	15.4 ± 0.5
Neutral detergent fiber	37.4 ± 0.8	36.3 ± 1.0	37.8 ± 1.5
Forage neutral detergent fiber	24.6 ± 1.3	29.0 ± 1.5	26.2 ± 3.0
Nonfiber carbohydrates	40.9 ± 1.1	40.5 ± 0.8	37.3 ± 3.1
Starch	24.1 ± 0.4	25.3 ± 0.7	23.9 ± 1.4
Ether extract	3.1 ± 0.1	3.5 ± 0.1	3.0 ± 0.1
Nutrition requirement model[2]	CPM	CPM	NRC

[1] ND and S farm used the same TMR purchased from the cooperative organization of farmers.
[2] NRC, National Research Council, 2001 model; CPM, Cornell-Penn-Miner dairy model.

TABLE S7 Examination of the statistical method used for the correlational analysis of microbiota data at the genus level with respect to AI frequency to conception using the DAtest software

Method	AUC	FPR	FDR	Power	Score
Spearman correlation coefficient	0.81	0.04	0	0.33	0.1
Log-linear regression model	0.75	0.04	0	0.13	0.03
Quasi-Poisson generalized linear model	0.86	0.05	0.23	0.67	0.01
Pearson correlation coefficient	0.86	0.05	0.21	0.6	0
Linear regression model	0.86	0.05	0.21	0.6	0
Negative binomial generalized linear model	0.93	0.16	0.6	0.87	–0.22

Because of AUC, FPR and Power, the quasi-Poisson generalized linear model was selected as an optimal statistical method for the microbiota data at the genus level.

TABLE S8 Examination of the statistical method used for correlational analysis of predicted functional ortholog data with respect to AI frequency to conception using the DAtest software

Method	AUC	FPR	FDR	Power	Score
Spearman correlation coefficient	0.88	0.04	0.04	0.65	0.21
Pearson correlation coefficient	0.9	0.04	0.11	0.79	0.2
Linear regression model	0.9	0.04	0.11	0.79	0.2
Quasi-Poisson generalized linear model	0.9	0.04	0.13	0.8	0.18
Log-linear regression model	0.87	0.04	0.1	0.65	0.14
Negative binomial generalized linear model	0.9	0.07	0.21	0.75	0.1
Poisson generalized linear model	0.87	0.48	0.9	0.86	–0.58

Because of AUC, FPR and Power, the Pearson correlation coefficient was selected as an optimal statistical method for the predicted functional ortholog data.
